# Perceptions of overweight and obesity among Chinese immigrants in Melbourne, Australia: A qualitative study

**DOI:** 10.1016/j.jmh.2026.100458

**Published:** 2026-10-30

**Authors:** Yixi Wang-Chen, Nicole J Kellow, Hui Yang, Tammie S T Choi

**Affiliations:** a Department of Nutrition, Dietetics & Food, Monash University, Melbourne, 264 Ferntree Gully Road, Notting Hill VIC, 3168, Australia; b Department of General Practice, Monash University, Melbourne, 553St Kilda Road, Melbourne VIC, 3004, Australia

**Keywords:** Migration, Obesity, Perception, Body image, Stigma, Qualitative research, Chinese diaspora, Acculturation, Culturally safe care

## Abstract

**Background:**

Chinese immigrants living in Australia navigate multiple cultural and social contexts. Their understanding of overweight and obesity may be shaped by migration experiences, family expectations, socioeconomic circumstances, media exposure, and interactions with healthcare systems. Examining these perceptions is key to developing culturally appropriate interventions.

**Method:**

This study adopted social constructivism as its philosophical paradigm. Semi-structured interviews with photo elicitation were employed to collect data regarding participants’ beliefs, thoughts, lay knowledge, and attitudes towards obesity. Participants’ eligibility criteria were 1) Self-reported Body Mass Index ≥23; 2) Self-identified as ethnic Chinese adults living in Melbourne, Australia for at least six months; 3) Fluent in either Mandarin or English. All interviews were audio-recorded and transcribed verbatim. Reflective thematic analysis was used for data analysis.

**Results:**

Twenty-five (13 females, 12 males) participants were interviewed either in person or virtually. Participants were from Mainland China, Malaysia, Hong Kong, Singapore, Indonesia, and Taiwan. Four themes were generated: (a) The body of “others” as a comparative reference; (b) Obesity as an aesthetic and moral obligation to change; (c) Commentary from others as self-monitoring; (d) Weight loss is a personal matter, and willpower is the key for success.

**Conclusion & implications:**

This study shows how a diverse Chinese diaspora living in Melbourne make sense of overweight and obesity through cultural logic shaped by family expectations, social norms, and their migration experiences. The findings highlight the importance of understanding weight through Chinese cultural meanings rather than solely applying Western interpretive frameworks. Such insights can inform culturally safe care that respects both individual narratives and the intersecting cultural contexts in which they are formed.

## Highlights

• The perceptions of overweight and obesity among the Chinese diaspora in Melbourne were shaped by the core values of Chinese cultures and social norms while simultaneously being negotiated through migration experiences.
• Chinese immigrants living in Melbourne found their new environment more accepting of obesity than in their home counties, bringing psychological relief but possibly reducing weight vigilance.
• Chinese immigrants often interpret obesity-related comments from others as expressions of care. The Western concept of obesity stigma may require appropriate cultural adaptation.
• Highly valuing willpower for achieving weight loss can be a double-edged sword: it may enhance self-control and well-being but may also encourage unproductive behaviours.

## 1 Introduction

An individual’s perception of obesity is not fixed but is socially constructed through interactions (Galbin, 2014) and influenced by the sociocultural environment and personal experiences. Immigrants represent a distinctive population navigating multiple socio-cultural contexts. The Chinese diaspora living in Australia often draw on experiences, values, and understandings developed in their origin societies, while also adapting to new sociocultural environments. Australia and their countries of origin differ not only in cultural values but also in economic development, population density, language, religion, food environment, healthcare systems, and governance/political systems. Meanwhile, in today’s digital era, physical migration does not entail informational disconnection. Chinese immigrants have access to Chinese-language media platforms (e.g., WeChat, TikTok, and LINE) (Sun, 2024) and communicate with family/friend networks easily from their home countries while living in Australia. Their perceptions of obesity are likely to be distinct from both those of non-immigrant Chinese and from native-born Australians, due to the dual exposure to information and social-cultural environments.

The term “Chinese immigrants” in this paper refers to Australian permanent or temporary residents who migrated from other countries and consider themselves ethnic Chinese. Individuals born in Australia were not considered immigrants in this study. Importantly, the term “Chinese” is used as an ethnicity rather than a nationality (e.g., Chinese citizen) category in this paper. As the target group belonged to the broader Chinese diaspora in Melbourne, Australia, the terms “Chinese immigrants” and “Chinese diaspora” are used interchangeably where the context permits. According to the 2022 Australian Population Census and the 2021 Cultural and Ethnic Diversity Census. Australian Bureau of Statistics (2022), (2021), there were nearly 1 million members of the Chinese diaspora living in Australia, with 70–80% originating from Mainland China, Hong Kong, and Taiwan. Other Southeast Asian countries, including Malaysia, Indonesia, Vietnam, Singapore, and the Philippines, also contributed a significant number of Chinese immigrants (Australian Bureau of Statistics 2022, 2021). The majority of Chinese immigrants to Australia settled in major metropolitan cities, such as Sydney, Melbourne, and Perth. Live in Melbourne (2021); City of Perth (2021) They tended to settle in areas that have better access to quality education, economic opportunities, and well-developed infrastructure, as well as proximity to other Chinese immigrants and cultural networks. Lee et al. (2025) Chinese immigrant communities highly value educational achievement (Chen, 2019; Watkins et al., 2017). The 2022 Being Chinese in Australia survey found that 51% of Chinese Australians held a university degree, significantly higher than the rate among Australians overall (26%) (Lowyinstitute 2022). Except for English, Mandarin and Cantonese are two main variations of the Chinese language spoken at home in Chinese communities in Australia (Australian Bureau of Statistics 2021).

Obesity among the Chinese diaspora living in Australia deserves attention. In general, Chinese immigrants have a lower average Body Mass Index (BMI) compared to the Australian-born population (Menigoz et al., 2016). However, the accurate prevalence of overweight and obesity in Chinese immigrants remains unclear. Australia is a multi-ethnic country that uses the WHO BMI cut-off points (BMI above 25 as overweight, above 30 as obesity) for its regular population census (Australian Bureau of Statistics, 2022). This inevitably underestimates the prevalence of obesity among Chinese immigrants, given that the recommended Chinese (BMI above 24 as overweight, above 28 as obesity) or Asian BMI cut-offs (BMI above 23 as overweight, above 25 as obesity) are lower than the WHO cut-offs (Region WHOWP 2000; National Health Commission of the People's Republic of China 2024). Nevertheless, reference data from Mainland China showed that, in 2022, about one in two Chinese people were living with overweight or obesity, using the Chinese BMI cut-offs (Ting et al., 2024). The rate of overweight and obesity among Chinese immigrants in Australia might be comparable or even higher than those in Mainland China, since Asian immigrants in developed countries tend to gain unhealthy weight as they acculturate (Menigoz et al., 2016). More alarmingly, Asians have a higher risk of developing chronic disease at a relatively lower BMI compared to individuals of European descent (Hsu et al., 2015).

Despite growing concerns about obesity among Chinese immigrants, there remains a lack of research attention on this population. The lack of reliable data on obesity prevalence among this group in Australia suggests an overall lack of research on this population. The Australian Obesity Management Guidelines have acknowledged this gap, stating that there is a lack of evidence to guide clinical management specifically for culturally and linguistically diverse people (Department of Health and Aged Care 2024), including Chinese immigrants. Perceptions of obesity play a critical role in shaping individuals’ behaviours. For instance, weight misperceptions among adults living with overweight and obesity have been associated with a lower likelihood of interest in weight loss and physical activity (Duncan et al., 2011). Qualitative research methods allow for the in-depth exploration of people’s perceptions of obesity. However, qualitative evidence on this topic among Chinese immigrants is scarce. A systematic review conducted by the authors synthesising research investigating the obesity perceptions of Chinese people found that fewer than 20% of included studies utilised qualitative methods, and only one out of 53 studies involved a subgroup of Chinese immigrants in America (Wang-Chen et al., 2025). Most existing studies on this topic rely on cross-sectional surveys, a design that allows for rapid results across larger samples but limits deeper exploration of the phenomenon. Therefore, the current study adopted a qualitative approach to explore perceptions toward obesity among Chinese immigrants living in Australia. Insights into their obesity-related perceptions post-migration can offer valuable insights for culturally sensitive interventions.

## 2 Method

This qualitative exploratory study utilised a social constructivist approach to understand the perception of obesity. It employed semi-structured interviews with photo elicitation for data collection. The interview question guide consisted of 10 questions, including participants’ definition of obesity, the perceived contributors to obesity, their weight change history, experiences living with obesity, weight management journey, information sources, and their attitudes toward obesity-related comments. The research team consulted with members of the Weight Issues Network (a registered health promotion charity consultation group, providing voice and support for people living with obesity) (Weight Issues Network, 2024), an experienced bilingual Chinese dietitian working in Melbourne, and a Chinese immigrant living with obesity to check if the questions were appropriate.

Photo elicitation is a method involving the use of photographs/images to evoke deeper emotions, memories, and ideas. (Glaw et al., 2017). It enhances data richness and depth; (Kyololo et al., 2023) Incorporating photographs can make the interview more engaging for participants; Visual images can serve as a bridge to help participants express complex or abstract perceptions more easily (Tewahido et al., 2023).

The images were designed based on the findings of a previous systematic review (Wang-Chen et al., 2025), which explored perceptions toward obesity among Chinese adults living in and outside of China. Ideas/scenarios relating to obesity perception were formed and turned into images, assisted by ChatGPT DALL.E image generation (OpenAi 2025). YW trimmed and edited the images via Procreate software to ensure they represent the ideas clearly. (See Supplementary file 1 for the process of image creation). All the images were iteratively revised within the team. The final images were gender-based with the same content, 5 for male and 5 for female participants. (See Supplementary file 2 for the question guide and images) At the end of each interview, participants were invited to look at the five images and select one image that resonated most with them and explain why. They were clearly informed that they may agree or disagree with any of the messages suggested by the image.

Ethical approval for this study was obtained from Monash University Human Research Ethics Committee (Project ID:43,387).

### 2.1 Purposeful sampling strategy

The target population of this study was Chinese immigrants living in Australia. This group is defined as self-identified Chinese individuals who were born and lived outside of Australia, later migrated to Australia. The participant inclusion criteria were permanent/temporary Chinese adult immigrants living in Australia for at least six months, BMI≥23, and fluent in either Mandarin or English.

In order to collect a diversity of experiences and reflect different perspectives, a maximum variation strategy was utilised. Creswell and Poth (2016) Participants were recruited through purposive sampling with maximum variation by age band, gender, country of origin, length of residence in Australia, education level, employment status, and BMI. The research team recruited participants exclusively from Melbourne. Since Melbourne is the second-largest city in Australia and is home to 30% of Chinese Australians (Lowyinstitute, 2022). Also, the research team is based in Melbourne and aims to conduct more in-person interviews, which tend to yield higher-quality interactions.

Information power was also considered. The concept of information power was developed by Malterud et al., who proposed that the richer the information provided by the sample, the smaller the sample size needed (Malterud et al., 2016). In this study, the richness of information relied on the quality of interviewing data, which was assessed by the interviewer. For example, if the participant only provided simple responses about the topic, the quality of the interview data was considered to be poor. The researcher would then look for more participants of a similar background to interview. In this study, Sunders et al’s discussion of saturation in qualitative research informed the data collection and analysis process, which proposed that saturation can be understood as when researchers become “confident of having achieved or at least closely approached thematic saturation” ((Saunders et al., 2018), p.1901). As data collection and analysis proceeded concurrently, the research team regularly met to discuss the emerging findings. Initially, a wide range of potential patterns were identified, but it was neither possible nor necessary to pursue all patterns. Instead, we made analytic choices about which directions were worth developing further. These choices were guided by the research questions, the novelty and relevance of the patterns, and the team’s assessment of which lines of inquiry would yield the most meaningful insights. By the 15th interview, a set of 4–5 themes had taken shape. This number was determined pragmatically based on the typical length of qualitative journal articles. Braun & Clark suggested that 2–6 themes are appropriate for a single journal article, as discussing more than six themes in sufficient depth is difficult within typical journal word limits (Braun and Clarke, 2019c). As additional interviews were conducted, incoming data only reinforced, rather than expanded, the thematic structure. After the 25th interview being conducted and analysed, we were confident that the constructed themes interpreted from the data can tell “a compelling, coherent, and useful story” (Braun and Clarke, 2019b)to address the research question.

### 2.2 Participant recruitment

The recruitment strategy utilised three approaches. Firstly, the recruitment bilingual e-flyers (English version, Traditional and simplified Chinese versions) were posted on multiple social media platforms, including WeChat, RedNote, and Facebook. Secondly, community organisations (e.g., Chinese language schools, clinics, public libraries, community centres, religious institutes, sports clubs, Asian grocery stores) were contacted. The recruitment flyers were either circulated in their newsletters or posted on their noticeboards. Thirdly, snowball sampling was adopted after ten interviews. The participants were asked to make a referral to their friends or colleagues to consider participating in the research.

### 2.3 Data collection

Interested individuals completed a screening questionnaire that collected their demographic data, self-reported height and weight, educational level, length of residence in Australia, age band, language spoken at home, and other relevant variables. The research team selected individuals based on the eligibility criteria and maximum variation strategy. Participants’ estimated BMI were calculated using their self-reported weight in kilograms divided by height in metres squared (kg/m²). This study adopted the Asian-pacific BMI classification, deeming a BMI above 23 kg/m^2^ as overweight and above 25kg/m^2^ as obesity (Region WHOWP, 2000). Eligible participants were contacted to schedule interviews. The interview language was either Mandarin or English. Informed consent was gained verbally before each interview. The interview began with the semi-structured questions, followed by the photo elicitation section. Participants received a AUD 30 grocery gift card as a token of appreciation. All interviews were audio recorded.

### 2.4 Transcription and translation

All the interviews were transcribed verbatim by YW. Staying as long as possible in the original language can reduce the potential semantic loss and preserve cultural meanings (Temple and Young, 2004). In this research, all the transcripts were transcribed in their original language. During data analysis, researchers coded the data in their preferred language independently. In the discussion meetings, researchers communicated in English. When presenting quotes in the result section, both the original quotes and the English translation were presented. These excerpts were translated into English using a meaning‑based approach. Three bilingual Chinese researchers reviewed all translations and resolved any differences through discussion to ensure accuracy and preserve culturally specific meanings.

### 2.5 Data analysis method

Reflexive thematic analysis (TA) was employed (Braun and Clarke, 2019a). It values the centrality of researcher subjectivity and reflexivity and considers the researcher as an active meaning-maker (Braun and Clarke, 2019a). Reflexive TA emphasises that themes are interpreted and generated by the researchers rather than emerging from the data or pre-existing in the data (Braun and Clarke, 2019a). Member-checking was not utilised in this study. The following steps described the data analysis process.

Firstly, YW familiarised herself with the data and generated initial codes using an inductive, data-driven approach. Open coding was applied to capture as many potential patterns as possible, with both semantic (descriptive) and latent (interpretive) codes generated. Coding was conducted using QSR-Nvivo (Version 14.23.0). Meanwhile, the three additional researchers independently analysed 10 randomly selected transcripts. Secondly, candidate themes were constructed by clustering recurring patterns around central concepts. Thirdly, team discussions were held regularly to review patterns and refine themes. Peer debriefing was conducted to seek feedback from colleagues and friends with diverse cultural backgrounds. These exchanges helped challenge potential cultural assumptions and analytic blind spots. Lastly, final themes were finalized based on their cultural relevance and practical implications. (The photo elicitation process generated textual data that was similar to the interview data. Therefore, the data analysis method for both the interviews and photo elicitation data was the same.)

## 3 Researcher positionality

YW is a bilingual, ethnically Chinese woman who grew up in China and moved to Australia to undertake tertiary study. She has not personally experienced living with obesity. This dual positioning makes her both an “insider” and an “outsider” among the research participants. She acknowledges that her values, professional training in public health nutrition, and personal identity inevitably influence how she interacts with participants and interprets their narratives. Her approach reflects a reflexive stance, which stresses self-examination while remaining attentive to participants’ perspectives. She is guided by a belief in critical cultural relativism (Nickerson, 2023) that every culture should be understood within its context. She is particularly cautious about the uncritical adoption of Western ideals in the realm of obesity research, which can obscure the value of non-Western traditions.

Additional members of the research team include two bilingual researchers (TC and HY) and one English-speaking researcher (NK). TC (female) and HY (male) are Chinese immigrants living in Australia, originating from mainland China and Hong Kong. TC and HY are experienced qualitative researchers with expertise in dietetics and public health. Complementing this, NK (female), an Australian nutrition researcher of Caucasian background, offers a Western and “outsider” viewpoint for this research.

The positionality of the research team shaped the analysis through a combination of emic and etic perspectives. As three members are bilingual ethnic Chinese researchers, their emic understanding of cultural norms, traditional values, and social expectations, as well as their varied degree of acculturation to Australian society, supported the use of a cultural relativism lens. This lens enabled the team to embrace their cultural knowledge to explore the underlying logic of participants’ accounts, rather than solely applying Western frameworks to critique Chinese values and practices. The etic perspective was contributed primarily by the English-speaking researchers, who encouraged the team to consider how culturally normalised beliefs might be beneficial or harmful for weight management and psychological well-being. This emic–etic dialogue informed coding decisions, interpretation, and the presentation of culturally sensitive findings.

## 4 Results

Ninety-seven individuals completed the initial screening form. Twenty-five eligible participants were invited for interviews. All (13 females and 12 males) completed the interview during August-November 2024. There were 18 in-person and seven online interviews. Sixteen interviews were in Mandarin, and nine were in English. The combined interview time was 23.6 h. Each interview lasted around one hour. Over half (56%) of participants originated from Mainland China, the rest were from Malaysia, Hong Kong, Singapore, Indonesia, and Taiwan. Nearly 70% of the participants self-reported having attained a university-level education or higher. The sample included participants across five age categories (18–29 years, 30–39 years, 40–49 years, 50–59 years, and>60 years), with representation in each category. Exact age was not collected due to cultural considerations. Participants had diverse employment backgrounds, including unemployed individuals, full- and part- time workers, self-employed individuals, university students, and retirees. The length of residence in Australia ranged from one to 36 years. Approximately 60% participants reported having no known chronic medical conditions, and the remaining 40% reported having one or more chronic diseases. Over half of the participants spoke Mandarin at home, followed by English, Shanghainese, and Cantonese. According to the self-reported body weights and heights, 92% of participants were categorised as obese, and 8% were overweight. (See Supplementary file 3 for detailed demographics). Regarding photo elicitation, the distribution of selected images was: Lonely journey (n = 4), Round table (n = 7), Motivation (n = 11), and Information (n = 2). Participants’ responses to the images were consistent with their responses to interview questions, suggesting that the images facilitated reflection of the reported experience and narratives.

Four themes were generated, with each addressing a domain in the perception of obesity: how obesity was defined, attitudes toward obesity, beliefs about weight management, and interpersonal interactions. Each theme begins by identifying culturally rooted patterns and then highlights the distinctive post-migration perceptions.

**a. The body of “others” as a comparative reference**

When asked how participants defined obesity, most reported that they knew or had heard of BMI and regarded it as a scientific tool for assessing obesity. Although participants reported an awareness of BMI, many still appeared to assess their weight status by comparing their appearance with that of others. Based on participants’ accounts, these “others” often included peers, people in their immediate environment, online figures, strangers in society, and a more abstract social construct: the so-called “正常人” (“normal person”). Our interpretation is that “normal person” refers to someone who does not carry excess weight and is perceived to have a well-proportioned body figure. As one male participant shared: [肥胖]就是比正常人胖嘛, 比正常人体重重, 然后看起来比较臃肿. (Obesity is someone who is bigger than a normal person, heavier than average, and looks quite bulky) — P11.

The frequent use of the term “正常人” (“normal person”) or “正常体重” (“normal weight”) among participants may suggest an innate expectation to conform to perceived societal ideals. This comparative mindset is likely to lead participants to categorise their weight as “abnormal”. Some participants even alienated themselves as “abnormal people”. From this perspective, the perceived sense of abnormality could add a layer of anxiety among female participants. A quote from a young female university student may reflect this point: 为什么我是这样子呢? … 其实我会更有一点自卑的感觉, 就是只有我这样 (Why am I like this?… To be honest, it makes me feel somewhat less confident, like I’m the only one [not fitting into the societal norm]. )—P01 Notably, male participants in this study generally did not express the same level of insecurity about appearance. Instead, they appeared to be more concerned about the health risks associated with obesity.

Following migration to Australia, exposure to different cultural norms appeared to shape participants’ reference points of understanding obesity. It appeared that the local Australians became the new visual reference for body size. Participants reported that they saw more large-bodied individuals in Australia compared to their home countries in Asia. This exposure appeared to ease personal anxieties about weight for many. Some participants reconsidered their weight status, from "overweight" or "obese" by Asian standards to "normal" in the Australian context. For example: [澳洲]很多人比我肥, 我自己不觉得自己非常肥, 但是在中国来讲, 我这种算是比较胖的吧. (Many people here [in Australia] are bigger than me, so I don’t consider myself very fat. But in China, I’d probably be seen as fat.) —P08; 在澳洲这边, 我感觉还挺正常的.我回[中]国去…我确实是属于超重了 (In Australia, I feel like my weight is quite normal. When I returned to China…I am surely overweight.) —P06

**b. Obesity as an aesthetic and moral obligation to change**

Participant narratives suggested a set of recurring societal norms and attitudes toward obesity. Based on participants’ accounts, obesity appeared to be widely perceived as unattractive, unacceptable, and morally problematic. Meanwhile, individuals with excess weight were often blamed and expected to make active efforts to lose weight. These perceptions were evident in how participants described the appearance of people living with excess weight, expressed obesity-related concerns, and articulated their motivations for weight loss. For instance, participants tended to draw on aesthetically loaded descriptors such as 臃肿(flabby), 肥硕(corpulent), 笨重(clumsy and heavy), 大腹便便 (paunch) to refer to people with larger bodies, including themselves. A participant offered a reflection on aesthetic norms: 从外观来看, 当然人喜欢瘦一点, 情愿偏瘦也不要偏胖 (when it comes to looks, people surely prefer to be slim, better to be slightly slim than fat) —P24. Another participant articulated his views with a noticeable moral undertone: 你如果有一点知识层次的话, 你肯定要自己要一点一点要改变嘛…因为你太肥胖的话, 你就是要浪费公共资源嘛(If you have a bit of civil responsibility, you would naturally start to change little by little… If you are too obese, you’re seen as a burden on public health resources)—P02. One participant even drew a connection between body weight and self-worth: being fat is also admitting that you are not as good as the other people. —P25 Overall, the responses indicated that participants were generally aware of their excess weight status, and their accounts suggested a tendency to align with prevailing social expectations about weight, with limited explicit questioning of these norms.

The migration experience appeared to encourage reflection on Chinese body culture and to open alternative ways of thinking. Participants commonly described experiencing a greater sense of psychological ease and belonging in Australia. For instance, many participants perceived Australian society as more accepting and less judgmental toward individuals with larger body sizes, compared to Asian societies. Some female participants reported changes in their attitudes toward body image through interactions with local Australians and everyday observations. They expressed feeling to some extent more confident about their bodies, less preoccupied with weight, and less burdened by constant comparison. The following quote illustrates how participants described experiencing this feeling:

国内就, 就是家里人会要求你美…对你外貌的这种规训 会比较多.所以我觉得到澳洲之后, 会让我觉得心里面更自在一点…我感觉更被包容."In China, especially within the family, there tends to be more pressure to look a certain way—to be 'beautiful.' There’s a kind of discipline around appearance. After coming to Australia, I felt a sense of ease… like I was more accepted and embraced for who I am." —P12

The ability to shift from being a passive recipient of body-related anxiety to a critical reflector on body culture may be partly associated with participants’ level of English proficiency. Participants with higher self-reported English fluency often appeared more able to engage with alternative discourses, such as body positivity or weight stigma critique. In contrast, older participants with limited self-reported English proficiency tended to reference more traditional views on obesity. However, English proficiency was self-reported rather than formally assessed.

**c. Commentary from others as self-monitoring**

Commenting on others’ weight appeared to be a common occurrence in participants’ interactions with close Chinese social networks. It seemed that body weight/size discussion was not considered a particularly sensitive subject in Chinese culture. The data showed that many participants not only felt it natural to receive weight-related comments, but they also regularly critiqued others on their body weight/size. On the other hand, these comments could be interpreted as being delivered in a pragmatic, outcome-oriented manner because comments often referenced increasing social capital (e.g., improving chances in employment or romantic relationships) or criticising individuals by not fitting in. Some comments were direct, abrupt, and at times appeared somewhat harsh. For example,

我妈在任何场合都会讲, 说你看大街上那个, 你看, 都这么瘦, 然后现在都没有胖人了, 然后你这么胖, 你觉得, 那意思是, 你, 你出去不会觉得就心里不舒服吗༟(My mom would say it on any occasion, like, "Look at the people on the street, they're all so slim. There aren't any fat people anymore. But you're so fat — don't you feel uncomfortable when you go out?") —P06

过年的时候,亲戚总是会说, 哎呀, 你应该减减肥, 你都头20岁了, 你马上要找对象结婚了.(During the New Year, relatives always say, Oh, you should lose some weight. You are already in your 20 s, and you will be looking for a partner to get married soon.) —P12

Interestingly, a few participants also reported initiating similar comments to others with the intention of showing care:

我有几个朋友, 其实他们也没怎么胖, 140斤, 我每次见到, 就说去你要减肥去了, 哈哈, 太肥了.(I have a few friends who aren’t really that fat, around 70 kg. Every time I see them, I say, you should go lose some weight. Haha, you are too fat.)—P03 comment to others

The majority of participants indicated that they did not experience these comments as hurtful, and some appeared to appreciate them as an expression of care. For instance: 她们说“你要减肥, 你的肚子好大欸” 人家说是为你好. (They say, you need to lose weight, your belly is so big. They say it for your own good.) —P03; 我希望他[朋友]诚实的跟我说, 我最近有没有胖还是瘦…因为是朋友, 无所谓的.(I want my friend to honestly tell me if I’ve gained weight or lost weight recently…I will feel okay with it because they’re my friends.) ——P04. For some, these comments could serve as reminders to be more mindful of their weight. A young male participant even considered such external feedback as a key part of his self-monitoring. As he said: 我对自己[体重]的评价主要来源于生活中别人对我的评价(My evaluation of my weight is mainly shaped by how others around me comment on it in daily life.)—P15. Others found the comments motivational: I felt motivated, and it was not annoying. Because what they are saying in reality, it is true, I did not take it as annoying, I took it as motivation. ——P19. However, there were notable exceptions. A few female participants reported frustration with repeated comments about their body weight and preferred such remarks to cease. For example: 我希望他们不要提[体重]…他们那评论有时候弄到我不是很comfortable, 虽然是善意的. (I wish they wouldn't comment on [my weight] … Sometimes their comments make me feel uncomfortable, even though they're well-intentioned) ——P16 This perception was observed primarily among female participants with higher BMI (e.g., BMI >28) and was rarely mentioned by other female and male participants. This may reflect the influence of gendered body norms, duration of living with obesity, family expectations, and obesity severity within Chinese communities, which often place greater emphasis on women’s body appearance.

Despite living in Australia, some participants tended to interpret weight-related daily interaction with Westerners through cultural norms from familiar Asian contexts. Many participants frequently reported that Westerners rarely commented on others’ weight, compared to Asian individuals. Rather than interpreting this as weight being a private or sensitive topic in Western society, some participants understood it as Westerners either not caring about them or not being close enough to comment on such personal matters. For example: [别人不评论], 大概是出于礼貌, 另外一个理解就是说, 你的胖, 关我屁事; (Others not commenting is probably out of politeness; Another way to interpret it is: Your fat ain’t my damn problem. )—P21

The absence of weight-related commentary could be a double-edged sword. Due to culturally ingrained expectations, some participants, especially males, relied on external comments as a form of weight self-monitoring. Meanwhile, several male participants shared that they rarely weighed themselves and had not known their weight for a long time. The lack of external feedback may weaken their awareness of weight gain or may lead them to neglect their overweight status. On the other hand, for those who were more sensitive to comments about their body, this absence could be experienced as psychologically beneficial, as it allows them to be less preoccupied with their weight.

**d. Weight loss is a personal matter; Willpower is the key**

A recurring pattern throughout the majority of participants was the view that weight loss is a personal matter, with self-discipline viewed as the key to success. They generally preferred self-initiated lifestyle changes over weight-loss medications or surgical interventions. Moreover, lifestyle change was understood as primarily involving adjustments to diet and exercise. This belief may partly explain why most participants felt they did not need professional assistance. The data suggests that most participants had no experience of visiting professionals specifically for weight issues. Only a few had consulted healthcare professionals (e.g., general practitioners, dietitians, fitness instructors). However, a common perception was that the advice received tended to be either generic or unsustainable. For some, the cost of visiting professionals was a barrier. Two dominant perspectives were summarized based on the data in this regard: first, that it was not worth spending money on something considered a personal responsibility; and second, that paying professionals for information freely available online was unnecessary. The following quotes illustrate these viewpoints:

还是感觉会浪费钱吧.你自己就可以瘦下来, 他们就是逼你做做事呗, 差不多, 那你就叫你父母逼着呗.(I still feel like it’s a waste of money. You can lose weight by yourself. Professionals are just pushing you to follow the plan, right? If that works, then you might as well ask your parents to push you instead) —P05

我觉得专业人士无非就帮我讲一些我可以搜到的东西, 我觉得有点浪费我时间(I feel like professionals are just telling me things I could find online myself. So, I think it’s a bit of wasting time. )—P04

Participants tended to view self-discipline/willpower as the key to successful weight loss and that enduring hardship was inevitable. Many participants expressed that the most difficult part of losing weight was maintaining “sufficient willpower” to adhere to their plans. They often blamed themselves for lacking self-discipline in response to repeated failures, while rarely reflecting on whether their dieting and exercise plans were programmatic, evidence-based, or sustainable. Furthermore, participants held varying beliefs about willpower/self-discipline. Some appeared to view it as an unlimited resource; if one truly made up their mind, success was seen as inevitable. As one participant noted:

因为就像我儿子这样, 他自己, 要想减肥的话, 肯定可以减成功的.(Like my son, if he really wanted to lose weight, he would definitely succeed) —P02 Others tended to believe that those who were able to maintain a healthy body weight were completely capable of resisting temptation. For example, 他[朋友]能做到每天吃东西, 只放点盐, 每天都吃水煮鸡胸肉, 我不可以.他吃泡面, 不放调料包. (He [a friend] can eat food seasoned with only a pinch of salt. He can just eat boiled chicken breast every day. I can’t. He eats instant noodles without the seasoning packet.) — P07. Some attributed their failed attempts to a lack of self-discipline: 我假如能够保持这样子[上次减重成果]就好了, 但是我的坚持太短了. (If only I had maintained my weight loss from last time. But my persistence was too short.) —P16

Another interesting pattern was that support from close others was typically verbal, but seldom translated into behavioural support. For instance: 父母说, 你很胖, 得减减肥啦, 到了吃饭还是说, 多吃点啊.(My parents say, “You are too fat, you need to lose weight”, but when it comes to eating, they still say, “Eat more”.)—P05; They (children) say, Mom, time to get up, do [it] yourself, you need exercise, like that. But if I ask them, can you walk with me? They say, Oh, not today, (laugh). —P14

## 5 Discussion

This study found that Chinese immigrants in Melbourne frequently drew on the bodies of others as comparative references for assessing obesity. Obesity was often understood through both aesthetic and moral frames. Obesity-related comments from others were often interpreted as expressions of care or kindly reminders. Many participants described weight loss as a personal responsibility, with willpower as the key contributor. The perceptions of overweight and obesity among the Chinese diasporas reflected both of relatively stable and dynamic features. They were influenced by the core values of Chinese cultures and social norms while simultaneously being negotiated through migration experiences.

**1. The moral obligation of obesity in the Chinese context**

Obesity seemed to be viewed differently through the Chinese lens among the Chinese community. Obesity stigma refers to negative societal attitudes and stereotypes about individuals living with obesity (Ramsamy et al., 2024). Drawing from this perspective, participants’ narratives in this study might appear to reflect the presence of pervasive obesity stigma within Chinese society. However, the term "stigma" implies a connotation of discrimination, devaluation, and denigration, which in Western contexts is seen as harmful and subject to elimination (Fulton et al., 2023). Whereas, this study revealed that many Chinese participants generally viewed the societal attitudes regarding obesity as a matter of fact and felt obliged to lose weight. The dominant pattern of perception among participants was not to question the legitimacy of “obesity stigma” but to discipline the self to avoid social marginalisation. Therefore, the Western concept of “obesity stigma” may not be directly applicable to the Chinese context.

Other evidence also lends support to this perspective. A systematic review of existing studies on obesity perception among Chinese adults, highlighted that there were only two out of 53 studies mentioning obesity stigma (Wang-Chen et al., 2025). A narrative review explored weightism in Asia suggested that there is a lack of awareness of obesity stigma in Southeast and South Asian countries (Mathialagan and Lau, 2024). The limited number of studies versus the large ethnic Chinese population may reflect the early stage of research development in this field, but it may also stem from “obesity stigma” being less relevant in contemporary Chinese societies. Secondly, the different obesity prevalence in society and history can foster different societal perceptions toward obesity. Taking China and Australia’s obesity histories as examples is particularly relevant, given that China is the largest source country of Chinese immigrants in Australia (Australian Bureau of Statistics 2022). In Australia, the obesity rate reached 19% in 1995 and continued to rise to 31.7% in 2023. (Nickerson, 2023), whereas China’s obesity rate in 1991 was 1.4% (Ramsamy et al., 2024) and reached 16.4% in 2023 (Fulton et al., 2023). The obesity epidemic in China has a much shorter history compared to Australia. Most individuals in China still appear to have a relatively smaller body size. This may shape Chinese people’s perception that obesity is controllable and likely to focus on promoting weight loss, rather than addressing anti-obesity stigma. This might be the similar situation for the Chinese communities in other southeast Asian countries such as Malaysia and Singapore. Furthermore, the 2024 Guidelines for the Diagnosis and Treatment of Obesity (National Health Commission of the People's Republic of China, 2024) and the 2022 Expert Consensus on the Prevention and Control of Obesity (Chinese Nutrition Soceity, Chinese Preventive Medicine Association, 2022) issued in mainland China made no mention of “obesity stigma”, but focused on why and how Chinese people should lose weight. The former document also emphasised the importance of enhancing the social adaptability of individuals living with obesity. (Braun and Clarke, 2021). In contrast, Australia’s National obesity Strategy 2022–2032 explicitly identifies the elimination of weight stigma as a shared social responsibility (Commonwealth of Australia, 2022). A potential explanation for this differing governmental discourse might be that the term “obesity stigma” was generated in Western evidence and translated into Eastern society, yet without appropriate adaptation, it could have less resonance in Chinese society.

Taken together, reducing obesity stigma in Chinese societies may benefit those who suffer silently from its psychological toll. However, simply translating the Western anti-obesity stigma framework into Chinese contexts may risk overlooking the cultural values and practical applicability. A more viable path may lie in careful cultural translation and gradual integration.

**2 Cultural logics behind the normalisation of body weight commentary**

According to our participant narratives, body weight did not appear to be a sensitive issue but a common topic in daily social interactions in Chinese society. This aligned with the three bilingual Chinese researchers lived experience as well. Weight remarks were often interpreted as well-intentioned reminders or expressions of care in Chinese context. Meanwhile, living in Australia, participants noticed that Westerners rarely commented on someone’s weight. This may stem from that body weight is increasingly regarded as a sensitive topic in Western societies. Unsolicited comments about another person’s body weight can be seen as intrusive and inappropriate (Chentsova-Dutton and Vaughn, 2012) This divergence of perceptions may not be a matter of politeness or social etiquette but rather reflect two fundamentally different cultural logics.

There are cultural dimensions which could partly explain how such comments are normalised in the Chinese social context. Firstly, Confucianism has long been embedded in Chinese societies as a guiding framework for social interaction (Park and Chesla, 2007). Chinese individuals are expected to fulfil relational obligations to maintain harmony in society (Markus and Kitayama, 1991). Also, East Asian cultures are characterised by an interdependent view of the self, which emphasises attentiveness to others and fitting in with the collective (Markus and Kitayama, 1991). This has led to a common phenomenon in Chinese societies that close family members like to intervene and comment “for your good”. Interestingly, this study showed that participants were not just receivers of remarks but also habitual givers, which also suggested a normalised culture of body criticism within the Chinese community. Secondly, self-esteem was observed to be different between the East (interdependent, collectivism) and the West (independent, individualism). In Chinese culture, the ability to adjust, restrain oneself, and maintain harmony with social context is considered to achieve self-esteem. In contrast, the ability to express oneself and validate internal attributes in Western society is to gain self-esteem (Markus and Kitayama, 1991). These underlying logics might explain why Chinese individuals have different perceptions toward obesity-related remarks from their Western counterparts.

The mismatched perceptions between Chinese and Western societies can lead to cultural misunderstandings for Chinese immigrants in Australia. As shown in this study, Chinese immigrants might interpret the absence of body-related comments by Westerners as a lack of love and care. On the other hand, their well-intentioned remarks on body weight could be seen by Westerners as rude or intrusive, leading to intergroup tension. Not recognising these cultural differences may trigger internal conflicts between Chinese immigrants and mainstream Australian society. It is important to note that participants’ tendency not to interpret obesity-related comments as stigmatising does not necessarily imply the absence of stigma. Among our study participants, attitudes towards obesity-related comments appeared to vary by both BMI and gender. Women with a BMI in the overweight range were more likely to interpret such comments as reminders or expressions of concern, whereas women with a BMI above 28 or more often described them as a source of pressure or distress. Gender differences were also observed. None of the male participants in this study expressed the view that obesity-related comments should be minimised; instead, they generally regarded such comments as understandable or acceptable. Future research is needed to further explore this issue. Nevertheless, the present study highlights that the meaning attached to weight-related comments may vary across cultural contexts and may not align with Western conceptualisations of obesity stigma.

**3 The double-edged sword perception of willpower/self-discipline**

This study showed a consistent perception among participants that willpower/self-discipline was viewed as the key to weight loss. This perception may carry certain advantages. For example, people holding this perception are likely to attribute their weight loss failure to their weaker willpower, which is an internal cause. A study found that higher internal weight locus of control was positively associated with psychological well-being in individuals living with obesity, (Keightley et al., 2011] aligning with other research linking self-control to subjective wellbeing (Massar et al., 2022] Therefore, in this study, this perception may not be inherently detrimental when taking psychological wellbeing into consideration. However, this perception could also lead to a chain of counterproductive behaviours. For example, many participants believed that their willpower is key and therefore considered professional help as unnecessary. They tended to rely on self-induced methods of weight control. These methods often involve extreme lifestyle changes, which were either abandoned quickly or led to quick but unsustainable weight loss. This could further reinforce their belief in willpower being essential. Following this line of reasoning, when weight is regained, people often attribute it to a lack of willpower rather than to the method being unsustainable or non-scientific.

Participants’ understanding of willpower also conflicted with research evidence. For example, they often viewed willpower as a personal trait, believing that with enough determination, they could persist and succeed. However, this lay belief appears to be inconsistent with Baumeister’s “ego depletion effect”, that willpower is the capacity to exert effortful control, which can be depleted, especially under emotional stress or fatigue (Baumeister et al., 2024). Also, participants tended to view willpower as fixed, with some individuals naturally possessing more than others. In contrast, evidence suggests that willpower functions more like a muscle, capable of being strengthened through certain types of training (Audiffren et al., 2022). Participants also believed that if they forced themselves to stick to a weight loss plan, regardless of whether it was enjoyable or not at first, it would eventually become a habit, and the suffering would fade. However, research shows that better self-control is related to less effortful resistance to temptation (Galla and Duckworth, 2015). Similarly, according to Skinner’s reinforcement theory, when a behaviour leads to unpleasant consequences, the behaviour will happen less frequently (Papageorgi, 2018).

Taken together, this study suggested a balanced view of willpower and self-discipline. Its empowering potential lies in providing individuals with a sense of self-control, which links to psychological wellbeing. However, overemphasis on self-discipline can lead to counterproductive behaviours. Participants' understanding of willpower in this study appeared to be intuitive and simplified, suggesting the need for further education or guidance.

## 6 Strengths

This study intentionally recruited a diverse sample of participants, including a balanced number of male and female participants. The interview question guide was developed through a rigorous process, which stemmed from the findings of a systematic review and was reviewed by different stakeholders. The use of semi-structured interviews with photo elicitation allowed richer and deeper data collection. The research team included members with both Western and Eastern cultural backgrounds. It provided multiple interpretive perspectives, ensuring a more comprehensive understanding of participants’ perceptions. As one of the few qualitative studies focusing on obesity perception among Chinese migrants in Australia, it contributes to a deeper and more nuanced exploration of this under-researched population.

## 7 Limitations

Despite efforts to recruit individuals with diverse migration histories and life experiences, the lead researcher’s background (female, from Mainland China) may have facilitated stronger rapport with participants from similar backgrounds. This familiarity may have shaped data elicitation and interpretation, although it also enabled culturally nuanced understanding. As participation was voluntary, the sample may have included individuals who were more willing to discuss weight-related experiences, which introduced possible self-selection bias. Participants may have been more inclined to share socially acceptable views and less willing to disclose more sensitive experiences, resulting potential social desirability bias. In addition, Melbourne was selected because it has one of the largest and most diverse Chinese immigrant populations in Australia, making it an appropriate setting for exploring a broad range of perspectives. However, experiences of Chinese migrants living in other Australian regions could differ due to differences in psycho-socio-cultural environments.

While migration-related factors (e.g., acculturation, migration stress, language barriers, discrimination, social isolation, and access to culturally safe services) may contribute to variation in obesity perceptions, this study only captured the broader perspectives of living with obesity post-migration. Future research may benefit from examining how obesity perceptions are shaped and reshaped by specific migration-related factors.

## 8 Implications

The findings suggest several implications for obesity care and public health practice. Given the widespread belief among participants that weight loss failure reflects insufficient willpower or self-discipline, public health messages may need to emphasise that weight management is a complex and long-term process. Educational efforts should focus on discouraging unrealistic expectations on rapid weight loss and promote sustainable approaches to weight management via a cultural lens. In addition, weight management initiatives for Chinese immigrants may benefit from engaging family members and close social networks, as participants frequently reported receiving verbal encouragement but limited practical support.

## 9 Conclusion

This study provided an intertwined narrative that reflects how obesity-related perceptions were preserved, negotiated, and transformed among a diverse Chinese diaspora living in Melbourne, Australia. The findings highlight the need for culturally sensitive weight management services in this population group. Encouraging self-compassion and building supportive environments—at personal, professional, and policy levels—are essential steps toward more effective obesity interventions for Chinese immigrant communities.

## Data statement

The research transcript data for this study is unavailable for access as it includes sensitive or confidential information for participants.

## CRediT authorship contribution statement

**Yixi Wang-Chen:** Writing – review & editing, Writing – original draft, Methodology, Investigation, Formal analysis, Conceptualization. **Nicole J Kellow:** Writing – review & editing, Supervision, Methodology, Formal analysis, Conceptualization. **Hui Yang:** Writing – review & editing, Supervision, Methodology, Formal analysis, Conceptualization. **Tammie S T Choi:** Writing – review & editing, Supervision, Methodology, Formal analysis, Conceptualization.

## Declaration of competing interest

The authors declare that they have no known competing financial interests or personal relationships that could have appeared to influence the work reported in this paper.
