# Supplementary material for: Perceptions of overweight and obesity among Chinese immigrants in Melbourne, Australia: A qualitative study
Source: J Migr Health. 2026 Oct 30;14:100458. doi: 10.1016/j.jmh.2026.100458 (PMC13757503; doi:10.1016/j.jmh.2026.100458)
Supplement: Supplementary file 1 [file mmc1.docx]

**The process of generated ten images for photo elicitation**

**Step 1: Scenario development**. The five scenarios reflect everyday situations experienced by Chinese individuals living with excess weight. These scenarios were developed from our literature review of Chinese‑ and English‑language studies on obesity perceptions among Chinese adults, (1) supplemented by input from three bilingual Chinese researchers. (See Table 1) The team deliberately chose simple black‑and‑white line drawings rather than coloured photographs to avoid introducing unnecessary visual information.

| **Table 1 Image Name and Scenarios Description** | |
| --- | --- |
| **Image name** | **Scenarios** |
| 1 Round table gathering | A larger-bodied individual sits with family or friends during a traditional Chinese dining occasion. |
| 2 Information sources | A person is overwhelmed by various weight loss messages and feels confused. |
| 3 Motivation | The primary motivation for weight loss is to improve confidence and appearance, with health as a secondary factor. |
| 4 A lonely journey | Weight loss is an isolating and solitary journey |
| 5 Eating alone | A larger-bodied person is eating while working or watching television at home. |

**Step 2: AI-based image generation.** Each scenario description was used as prompt to generate simple black line illustrations using ChatGPT DALL.E image generation tool. (2) For Scenario 1, 4, and 5, Five varied images were generated per scenario. The most representative image was selected for further refinement. For scenario 2 and 3, only key individual visual elements were generated, which served as components for later composition.

**Step 3**: **Artistic refinement in Procreate**. The selected images and elements were imported into Procreate. The researcher manually refined each illustration via editing, removing, adding components to ensure the visual meaning aligned with the intended scenario. (See Table 2)

| **Table 2 DALL.E-Generated Illustrations and Researcher-Modified Final Images** | | | | |
| --- | --- | --- | --- | --- |
| **Image name** | **AI generated image and elements** | **Revised/Final images** | | |
|  |  | **For male participants** | **For female participants** | |
| 1 Round table gathering |  |  | |  |
| 2 Information sources |   |  | |  |
| 3 Motivation |  |  | |  |
| 4 A lonely journey |  |  | |  |
| 5 Eating alone |  |  | |  |

**Reference**

1.	Wang-Chen Y, Yang H, Kellow NJ, Choi TST. Exploring the perceptions of Chinese adults toward overweight and obesity: A systematic literature review. Obes Rev. 2025:e13913.

2.	OpenAi. DALL·E: Image generation from text prompts. 2025.
