# Supplementary material for: Perceptions of overweight and obesity among Chinese immigrants in Melbourne, Australia: A qualitative study
Source: J Migr Health. 2026 Oct 30;14:100458. doi: 10.1016/j.jmh.2026.100458 (PMC13757503; doi:10.1016/j.jmh.2026.100458)
Supplement: Supplementary file 2 [file mmc2.docx]

| Participant characteristics （n = 25） | |
| --- | --- |
| Participant characteristics | **Count (n)** |
| Gender | Males n = 12;  Females n = 13 |
| Age band (years） | <30 n = 6; 30- n = 8; 40- n = 3; 50- n = 4; 60 and over n = 4; |
| Origin countries or regions | Mainland China n = 14; Hong Kong n = 3; Malaysia n = 4;  Singapore n = 2;  Indonesia n = 1;  Taiwan n = 1; |
| Length of residence in Australia (years) | <3 n = 4;  3- n = 8; 10- n = 7; 20- n = 3;  30 and more n = 3; |
| Education level | Bachelor’s degree or above n = 17; High school n = 3; Vocational/technical qualification n = 5; |
| Employment status | Full-time workers n = 10; Part-time workers n = 7; Unemployed individuals n = 2; University students n = 3; Retirees n = 3; |
| BMI (kg/m²） | Overweight: 25 > BMI ≥ 23, n = 2;  Obesity: 28 > BMI ≥ 25, n = 7;  30 > BMI ≥ 28, n = 5;  BMI ≥ 30, n = 11; |
| Self-reported chronic conditions | No known chronic disease, n = 14;  One or two types of chronic disease, n = 10  More than two types of chronic disease, n = 1; |
| Family size | Living alone, n = 3;  Living with 1-2 people, n = 12;  Living with 3-4 people, n = 9;  Living with 5 and more, n = 1 |
| Self-reported English proficiency (1 = poor, 10 = fluent) | <3 n = 3  3- n = 10  7- n = 9  10 n = 3 |
| Language speaking at home | Mandarin, n = 14;  Shanghainese, n = 3;  Cantonese, n = 2;  English, n = 6; |
| Religion/belief | Buddhist, n = 5;  Catholic/Christian, n = 7;  Atheist, n = 6;  Agnostic, n = 1;  Spiritual, n = 1;  Prefer not to say, n = 5; |
| Annual personal income (AUD) | Living on pension, n = 3;  Less than 30 000, n = 5;  30 000 – 60 000, n = 1;  60 000 – 90 000, n = 10;  90 000 – 12000, n = 4;  Above 12 000, n = 2; |

**Footnote:** BMI = Body Mass Index; calculated as weight (kg) divided by height (m²); For employment status, participants selected the category they identified with most.
