# Supplementary material for: Perceptions of overweight and obesity among Chinese immigrants in Melbourne, Australia: A qualitative study
Source: J Migr Health. 2026 Oct 30;14:100458. doi: 10.1016/j.jmh.2026.100458 (PMC13757503; doi:10.1016/j.jmh.2026.100458)
Supplement: Supplementary file 3 [file mmc3.doc]

**Semi-structured interview question guide and photo elicitation images**

1 Let’s start with a general question. What is your definition/understanding of “obesity” or “overweight” ？ 让我们从一个最基本的问题开始。您如何理解肥胖或超重？

2 Think about an ordinary day, what occasions make you think about your weight? Or is body weight a concern in your everyday life? 回想平常的一天，您会在什么情况下想到体重问题或者觉得自己太重？（或体重问题是否是您生活里的困扰？）

3 After migrating to Australia, did your perceptions of obesity change? How? 来到澳大利亚生活后，您对肥胖的看法有改变吗？如何改变的？

4. In modern society, what factors do you think prevent people from maintaining a healthy weight? 在现代社会，您觉得什么因素让人们难以保持健康体重呢？

5. How has your weight changed so far? (prompts: childhood, adolescent, university, dating, marriage, childbirth) Tell me about your lightest and heaviest weight, what caused that? (e.g., medicine, sedentary job, losing loved ones) 您覺得您的体重变化情况如何？ （e.g.,童年、青少年、大学时期、约会、结婚、生孩子时的体重。 告诉我您到目前为止最轻和最重的体重是什么时候，是什么原因导致的, e.g., 药物？工作？亲人离开？）

6. Have you done anything to manage your weight? (If yes, what measures did you use, how do you think about it? Yo-yo dieting experience? what is the biggest barrier during your weight loss process? Or what kind of method attracts you? ) 您有采取过任何措施来控制的体重吗？（如果有，用了哪些方法呢？你如何看待这些方法？是否有反复复胖的经历？在减肥过程中最大的障碍是什么？或者哪种方法吸引了你？如果没有，为什么呢？）

7. At the moment, are you doing anything for weight control? If yes, what are the reasons? 目前，您在做任何控制体重的事情吗？为什么呢？

8 Have you experienced others making comments on your weight? On what occasions and in what way? How did it make you feel? 您是否经历过别人对你的体重发表评论？什么样的情况发生的呢？这样的评论让您有何感受？”

9 How did others’ comments make you feel? 他人的评论让您有何感受？

10 Do you actively look for information regarding weight management? If yes, where did you obtain them? If not, why? 您平常会积极的寻找关于减肥面的信息吗？如果会，减肥方法哪里获取的？

| **Photo elicitation: Images provided for participants** | | |
| --- | --- | --- |
| **Image name** | **Photos designed for male participants** | **Photos designed for female participants** |
| 1.Round table gathering |  |  |
| 2.  Information sources |  |  |
| 3. Motivation |  |  |
| 4.A lonely Journey |  |  |
| 5.Eating alone |  |  |

（Note: The images were initially generated with the assistance of a generative AI tool and subsequently modified by the author for research purposes.）
